# Surgical Treatment of a Rare Isolated Bilateral Agenesis of Anterior and Posterior Cruciate Ligaments

**DOI:** 10.1155/2014/809701

**Published:** 2014-08-13

**Authors:** G. Cerulli, A. Amanti, G. Placella

**Affiliations:** ^1^Istituto di Ricerca Traslazionale per l'Apparato Locomotore, Nicola Cerulli, LPMRI, Arezzo, Italy; ^2^Catholic University of Sacred Heart, Policlinico Gemelli, Rome, Italy

## Abstract

The isolated bilateral agenesis of both cruciate ligaments is a rare congenital disorder. A 17-year-old male came to our attention due to an alteration in gait pattern, pain, and tendency to walk on the forefoot with his knee flexed. The patient did not recall previous injuries. Upon physical examination anterior and posterior chronic instability were observed. Radiographic examination of both knees showed hypoplasia of the tibial eminence, a hypoplastic lateral femoral condyle, and a narrow intercondylar notch. MRI brought to light a bilateral agenesis of both posterior cruciate ligaments. Arthroscopic evaluation confirmed bilateral isolated agenesis of both cruciate ligaments. We recommended a rehabilitation program to prepare the patient for the arthroscopic construction of both cruciate ligaments.

## 1. Introduction

Agenesis of the cruciate ligaments is a rare congenital malformation. Its incidence is 0.017 per 1000 live births [1]. This deformity was described for the first time in 1956 by Giorgi [2]. The most common form of agenesis affects the anterior cruciate ligament, although cases have been described with agenesis of both cruciate ligaments and, recently, an isolated agenesis of the posterior cruciate ligament [3–8]. Generally, the malformation is unilateral [9–17], more rarely bilateral [5, 6, 14, 18]. In the literature most of the cases described showed that this condition is usually associated with other abnormalities of the musculoskeletal system, in particular of the lower limb such as agenesis of the menisci [19]; tibial spines [20]; agenesis or dysplasia of the patella, tibia, and fibula [11, 12, 18, 21, 22]; focal defects of the proximal femur [17, 22, 23] and multiple organ syndromes like thrombocytopenia-absent radius syndrome (TAR) [24, 25] and arthrogryposis [26]. A congenital defect of one or both cruciate ligaments determines well-defined morphological alterations of both the intercondylar notch and tibial spines, as can be seen upon radiographic examination of the “tunnel view” and the MRI [2, 13, 16, 17, 20, 22, 27]. Often, patients have adapted to the congenital abnormality, and instability occurs after a traumatic event [4, 6, 7, 18, 20, 24, 28, 29]. The congenital absence of cruciate ligaments causes chronic knee instability leading to biomechanical alterations of the knee that may cause associated lesions and finally osteoarthritis. In this paper we present an extremely rare case of bilateral agenesis of both cruciate ligaments without other associated congenital malformations.

## 2. Case Report

A 17-year-old male came to our attention reporting pain in both knees and the sensation of “something wrong” while walking. He tended to walk on his forefoot with his knees flexed.

The patient reported no history of chronic instability and no important functional limitation in either knee; he was able to perform all sport activities.

The patient was born at term, he was the second of three brothers, his development was regular, and he did not suffer systemic diseases. No abnormal findings were recorded. The patient did not report previous traumatic events.

Physical examination demonstrated a lack of joint effusion, with marked anterior and posterior laxity. Incompetence of the anterior cruciates was present, with a grade 3 anterior drawer and positive pivot shift. Incompetence of the posterior cruciates was present, with a grade 3 posterior drawer, posterior sag, and positive reverse pivot shift. Dial test was positive at 30 and 90 degrees of flexion. There was no varus or valgus instability at 0 and 20 degrees of flexion. There was evidence of hyperextension and slight varus alignment. The left knee demonstrated medial joint line tenderness on the McMurray test.

**Figure 1:**
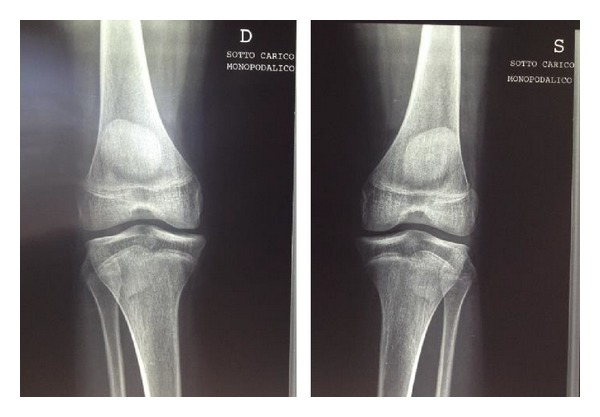
Anteroposterior view of the knees. Note the absence of the tibial spines.

**Figure 2:**
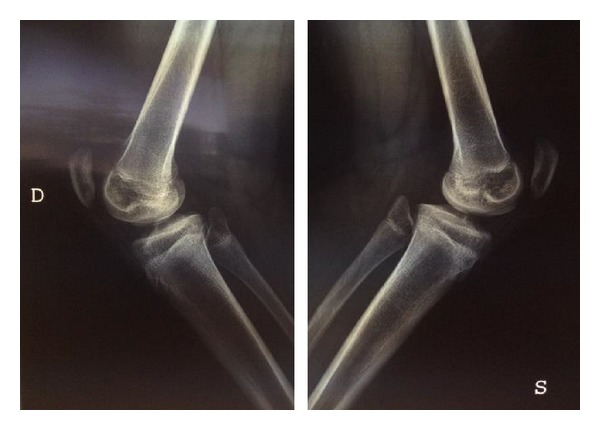


**Figure 3:**
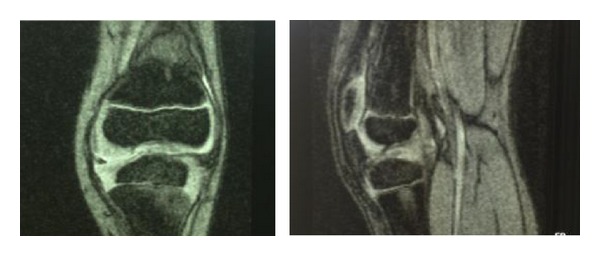
MRI of right knee.

**Figure 4:**
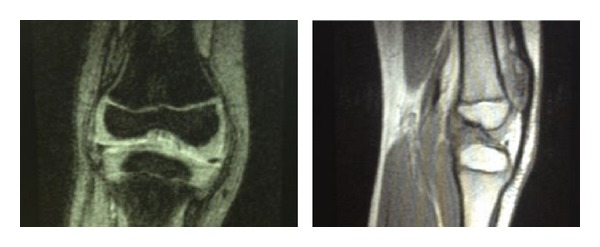
MRI of left knee.

Radiographic examination of both knees weightbearing showed a slightly varus knee, a narrow intercondylar notch, hypoplastic tibial spines, dysplasia of the lateral femoral condyles, and patella alta (Figures 1 and 2). MRI delineated the bony changes seen on plain radiographs, as well as a markedly narrowed intercondylar notch, flattened femoral trochlea, and hypoplasia of the medial facet of the patella. The menisci were intact, and the articular cartilage was uniformly thinned but intact. There was a complete hyaline cartilage covering the hypoplastic tibial eminence and a complete cartilaginous coverage of the shallow femoral notch in its anterior aspect. There was thickened synovium within the notch, where a ligamentous structure was recognizable, although irregular, with a trajectory consistent with a remnant of anterior cruciate ligament. A posterior cruciate ligament could not be identified. The collateral ligaments were intact (Figures 3 and 4).

Arthrometry with KT-1000 showed marked anterior laxity in both knees and a considerable posterior laxity. The measurement of muscular force by isokinetic evaluation registered a decrease in strength of 22% of the flexor muscles on the left side. The stabilometric assessment demonstrated a reduced proprioceptive control on the left side.

The patient underwent bilateral diagnostic arthroscopy to confirm the total lack of any structure of the central pivot under direct vision and assess the biomechanics of these very uncommon knees.

**Figure 5:**
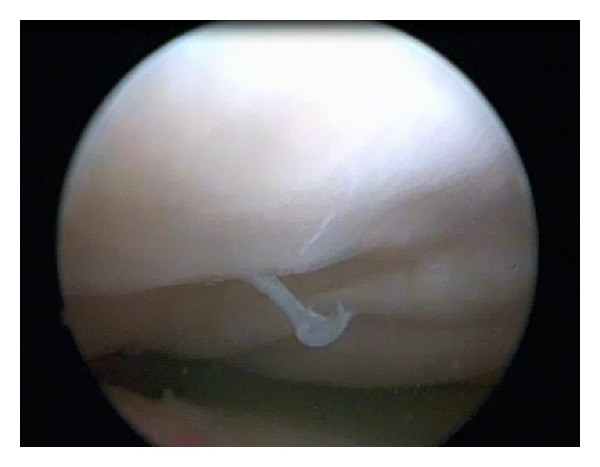
Arthroscopic view of right knee. Note the complete absence in the intercondylar notch of any structures of the central pivot.

**Figure 6:**
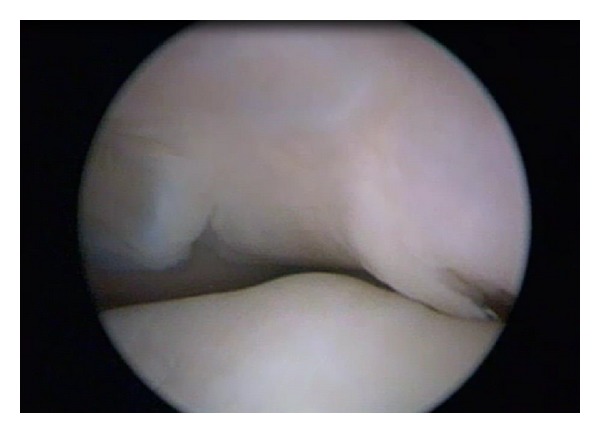
Arthroscopic view of the intercondylar notch of the left knee. The tibial spines covered by synovium tissue were deformed like a “ball-and-socket” joint.

The arthroscopic investigation confirmed the anatomical features of the intercondylar notch and tibial spines found on radiography and MRI. In fact the intercondylar notch was completely altered; it was small and completely occupied by synovial tissue (Figures 5 and 6). The anatomical areas of insertion of the cruciate ligaments were completely bare. In contrast with the MRI evaluation there was bilateral agenesis of both cruciate ligaments. In the right knee, we found a small cartilage defect of the medial femoral condyle. In the left knee we found a flap lesion of the posterior horn of the medial meniscus, which was treated with selective meniscectomy. We proposed conservative treatment consisting mainly of muscular strengthening and proprioceptive exercises.

After six months of conservative treatment, there were no improvements in terms of knee stability and pain relief; therefore plastic surgery of the intercondylar notch and both cruciates was proposed.

The first knee to be operated was the left one because of the better proprioceptive outcome. The hamstrings tendon was chosen for the autograft: quadrupled Gracilis tendon was used for ACL and quadrupled semitendinosus tendon for PCL. Thanks to the Original All-Inside Technique which uses half tunnels, short grafts, and manual drilling, we were able to perform all procedures during one surgery in a short time and in a tissue sparing manner.

After six months of rehabilitation and physiotherapy, we performed a clinical examination and biomechanical evaluation.

The patient had no left knee pain while walking or running and he relied more on his left knee than on the right one.

Therefore, we decided about two years after the first surgery to repeat the same procedure on the right knee.

**Figure 7:**
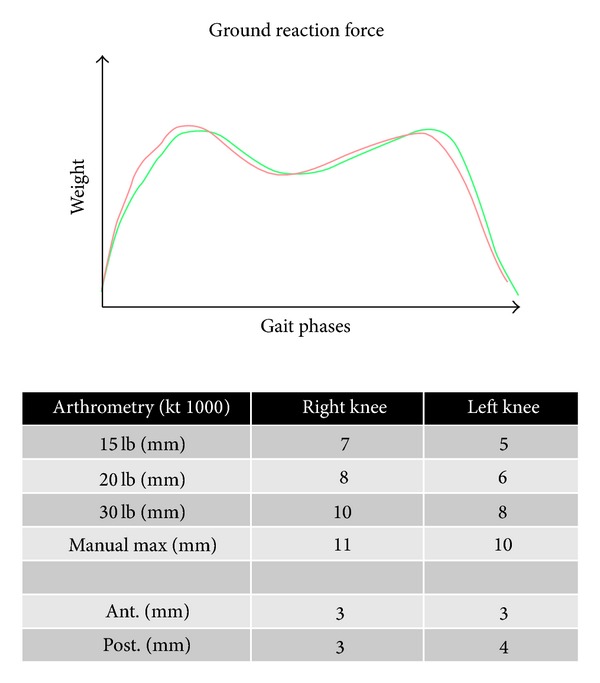
Biomechanical evaluation post-op. The new biomechanics created in the knees is similar to a normal knee with intact native cruciate ligaments.

After 8 months we performed clinical and biomechanical evaluations that revealed a greater stability at the arthrometric evaluation and at gait analysis; the patient no longer felt pain and began to run intensively and with increased self-confidence (Figure 7).

## 3. Discussion

Congenital deficiency of the cruciate ligaments is an extremely rare disease involving a deficiency of either the anterior or the posterior cruciate ligaments or both and may include one or both knees [30, 31]. Deficiency and dysplasia of cruciate ligaments of the knee joint are the main cause of congenital knee dislocation. The first suspected case of congenital absence of the cruciate ligament was reported in 1956 [2] and was later confirmed in 1967 by surgical exploration in patients with congenital dislocation of the knee [27]. Subsequently many authors have reported several cases of agenesis of the cruciate ligaments, in most cases, associated with other congenital malformations, such as deformity of the meniscus [9, 19, 24], flat tibial spine [2, 20, 28], shallow femoral intercondylar notch [17], femoral condyle dysplasia [17, 22, 23], valgus knee, fibula dysplasia, dysplasia of the patella [11, 12, 18, 21, 22], hip dysplasia, and idiopathic scoliosis [31]. Rare reports associate this disorder with multiple congenital abnormalities in other parts of the body, usually syndromic conditions such as thrombocytopenia-absent radius syndrome (TAR) [24, 25] and arthrogryposis [26].

Patients with agenesis of the cruciate ligaments often do not complain of joint instability because they usually adapt easily to the pathological anatomical condition [6, 7, 24]. Commonly laxity occurs after a traumatic event [5, 6, 18]. In these patients, the physician must always objectively differentiate the laxity assessed by the positivity of clinical tests and the patient's perception of instability [4, 28, 29]. Normally, it is difficult for radiologists to distinguish between traumatic and congenital causes of an absent cruciate ligament. There are several clues that may suggest one or the other. History of trauma to the knee suggests a traumatic cause. For children under the age of fourteen, injuries to the cruciate ligaments are less likely to be seen since the physeal plates are not yet fused and traction forces are more likely to cause epiphyseal separation, long bone fractures, or avulsions of the tibial eminence rather than a disruption of the ligaments [13, 32]. X-rays show several radiological signs that indicate the absence of cruciate ligaments such as hypoplasia of the tibial eminence [20, 22, 27], a hypoplastic lateral femoral condyle [16], or a narrow intercondylar notch [2, 13]. In a recently published study on the typical radiological findings of patients with arthroscopically proven aplasia of the cruciate ligaments, Manner et al. evaluated the associated pathological findings on MRI and tunnel view radiographs introducing a three-stage classification system [17]. They analyzed congenital dysplasia of the cruciate ligaments of the knee joint in 31 patients (34 knee joints) and classified this disease into three types. Type I includes hypoplasia or aplasia of the anterior cruciate ligament with a partially closed femoral notch and a hypoplastic tibial spine (Type 1 in 56%). Type II is characterized by aplasia of the anterior cruciate ligament and hypoplasia of the posterior cruciate ligament. In addition, the femoral notch and the tibial spine are worse than those of Type I (Type II in 21%). Type III knees have aplasia of both the anterior cruciate ligament and the posterior cruciate ligament with complete absence of the femoral intercondylar notch and aplasia of both tibial spines. According to the authors' results, the differentiation between trauma and aplasia of one or both cruciate ligaments may be made on the basis of differences in the notch width index and notch height and changes in the lateral and/or medial tibial spine [17].

The exact origin of this disease is still uncertain. Despite many studies on the development of the knee joint [33–37], the development of the cruciate ligaments is not yet clear. The cruciate ligaments are thought to be derivatives of the homogenous articular interzone [34, 38]. The exact time of their appearance differs according to the author [33, 34, 37–39]. It is well known that, between the seventh and tenth weeks of intrauterine life, knee structures are formed by direct condensation and differentiation of interchondral disk tissue [37]. According to O'Rahilly, cruciate ligaments may appear in stage 20 but it is more frequent in stage 21. They form the cellular condensation of the homogenous interzone [34, 38]. Chondrification of the femoral and tibial condyles begins in stage 20 and 21 while the formation of the joint cavity in the femoropatellar joint begins in stage 21. The cruciate ligaments are clearly visible and separated by a mass of loose connective tissue cells of the interzone. The different direction of the anterior cruciate ligament and the posterior cruciate ligament is observed. In a recent study, Ratajczak demonstrated the early appearance of the cruciate ligaments as well as the menisci in all investigated embryos. Both structures appeared in stage 19 as condensations of blastemal cells in the homogenous interzone [40]. The menisci, capsule, and cruciate ligaments all arise from this blastema, perhaps explaining why abnormalities in these structures commonly coexist [27]. In stages 22 and 23, Gardner and O'Rahilly [34] and O'Rahilly [41] observed the cruciate ligaments as distinct cellular condensation, with blood vessels around them. The posterior meniscofemoral ligament, the Wrisberg ligament, was not observed in our investigations. Only a few authors reported its presence at the 10th week and in 12.5 weeks [33, 37, 42, 43]. Some authors believe that the posterior cruciate ligament is the first to be formed, though in the literature there are no results concordant [34, 44]. The congenital anomaly that determines the anatomical defect is expressed around the seventh week to eighth week postovulation as described by Manner et al. [17]. Authors continue to debate whether the changes in the femoral intercondylar notch and the tibial spines are congenital or simply a secondary response to the aplastic cruciate ligaments [45]. The main role of the intercondylar notch seems therefore to be accommodating the cruciate ligaments; therefore if the ligaments are absent, the intercondylar notch fails to develop. Some authors state that the development of the intercondylar spine of the proximal tibia depends on the existence of the cruciate ligaments, so if the cruciate ligaments were congenitally absent, this would ultimately cause dysplasia of tibial spine [2]. Other authors consider that dysmorphism is caused by congenital dysplasia and not by a reaction to the agenesis of the cruciate ligaments because when the cruciate ligaments are completely absent, there is a consequent formation of the knee like a “ball-and-socket” where the intercondylar notch is completely covered with hyaline cartilage [17].

The congenital absence of both cruciate ligaments causes a condition of chronic instability of the knee, the long-term effects of which are not well known. The absence of the cruciate ligaments results in biomechanical alterations to the knee that may lead to meniscal lesions, chondral lesions, and eventually osteoarthritis, especially in the medial section [46–50]. Unlike primary osteoarthritis, the patellofemoral joint and the lateral compartment are less this affected. Cartilage degeneration in these patients occurs at an older age and with a slower evolution compared to subjects with traumatic ligament injuries. Some authors also report that the long-term outcome of knee instability due to the congenital absence of the cruciate ligaments is very good and many patients do not develop long-term degenerative changes [20].

Agenesis of the cruciate ligaments is a rare congenital malformation; usually this is associated with abnormalities of articular structures or systemic syndromes. In the literature, isolated bilateral agenesis of both cruciate ligaments is reported only in very few cases. Clinical, radiographic, and therapeutic approach in these patients are very difficult. In fact, in the literature, cases of misdiagnosis have been reported, such as confusing congenital instability with posttraumatic instability or radiographic, and MRI findings were misinterpreted [15, 30]. In our case, the MRI finding was confirmed both by the physical examination and by the arthroscopic evaluation.

In the literature there is controversy regarding the therapeutical options [30]. A good outcome has been observed after the reconstruction of cruciate ligaments in symptomatic patients with congenital absence of cruciate ligaments [4, 5, 15, 24]. Other authors prefer conservative treatment with physiotherapy and muscular training [3, 7, 11, 20, 28]. There are indeed patients who remain asymptomatic and are kept under observation. If surgical treatment is considered, it should include the reconstruction of both ligaments, since the reconstruction of the ACL alone results in posterior subluxation of the tibia and a fixed posterior drawer causing decreased knee extension and anterior knee pain [15, 29, 51].

Reconstruction of both cruciate ligaments is essential to restore knee stability and biomechanical function. Bilateral anterior and posterior cruciate ligament reconstruction as in the case we describe is technically challenging. The patient will need to undergo the reconstruction of both cruciate ligaments.

The reconstruction of both cruciate ligaments using autografts was made possible thanks to the use of the Original All-Inside Technique for ACL and by applying the same rationale for the PCL, that is, using the same drill, short grafts, and short tendon, thus sparing bone and soft tissues [52–54].
